# Epidemiology of Cutaneous T-Cell Lymphomas: A Systematic Review and Meta-Analysis of 16,953 Patients

**DOI:** 10.3390/cancers12102921

**Published:** 2020-10-11

**Authors:** Gabor Dobos, Anne Pohrt, Caroline Ram-Wolff, Céleste Lebbé, Jean-David Bouaziz, Maxime Battistella, Martine Bagot, Adèle de Masson

**Affiliations:** 1 Dermatology Department, Saint-Louis Hospital, AP-HP, 75010 Paris, France; gabor.dobos@charite.de (G.D.); caroline.ram-wolff@aphp.fr (C.R.-W.); celeste.lebbe@aphp.fr (C.L.); jean-david.bouaziz@aphp.fr (J.-D.B.); adele.demasson@aphp.fr (A.d.M.); 2 INSERM U976 Human Immunology, Pathophysiology and Immune Therapies, Institut de Recherche Saint-Louis, 75010 Paris, France; maxime.battistella@aphp.fr; 3 Department of Medicine, Université de Paris, 75010 Paris, France; 4 Institute of Biometry and Clinical Epidemiology, Charité-Universitättsmedizin, 10117 Berlin, Germany; anne.pohrt@charite.de; 5 Pathology Department, Saint-Louis Hospital, AP-HP, 75010 Paris, France

**Keywords:** cutaneous T-cell lymphomas, lymphomas, skin, mycosis fungoides, systematic review

## Abstract

**Simple Summary:**

Cutaneous T-cell lymphomas (CTCL) are rare malignant diseases. In this study we have compared the cutaneous lymphoma registries of different countries, which included information on at least 100 patients. The frequencies of each CTCL subtype were compared within and between continents. We found that the registries differed importantly in terms of size and quality. Some rare CTCL subtypes, such as NK/T-cell lymphoma or subcutaneous panniculitis-like lymphomas, were more frequent in Asian countries, while others were evenly distributed. We discuss possible reasons for this and provide suggestions on how to build future CTCL registries.

**Abstract:**

Cutaneous T-cell lymphomas (CTCL) are a heterogenous group of rare diseases. Many studies have reported on local epidemiology or geographic clustering, however we lack information from a global perspective. A systematic review and meta-analysis was conducted in Medline and the Cochrane Library based on a previously registered protocol and according to the preferred reporting of items for systematic reviews and meta-analyses (PRISMA). We selected publications that enrolled at least 100 patients with primary cutaneous lymphomas according to the current classifications. The relative frequencies (proportions) of subtypes were compared between studies and geographic regions in a meta-analysis. In total, 26 studies met our inclusion criteria, reporting on altogether 16,953 patients. Within primary cutaneous lymphomas, CTCL appeared to be 15% more frequent in Asian populations. Mycosis fungoides (MF) accounted for 62% of CTCL, with an important heterogeneity in frequencies between studies and continents. The proportion of Sézary syndrome (SS) was 3%, stable worldwide. Rare CTCL, such as NK/T-cell lymphoma or subcutaneous panniculitis-like lymphoma, were more frequent in Asian studies. This global meta-analysis of CTCL confirmed the predominance of CTCL among primary cutaneous lymphomas (83% on average) in the three analyzed continents, most of which were MF cases. It revealed the same proportions of SS across continents, and the heterogeneity of MF frequencies, suggesting the possible role of environmental factors in the pathophysiology of the latter. Registration number: CRD42020148295 (PROSPERO).

## 1. Introduction

Primary cutaneous lymphomas (CLs) are a heterogenous group of diseases belonging to the extranodal non-Hodgkin lymphomas. Contrarily to their systemic counterparts, primary cutaneous T-cell lymphomas (CTCL) are more frequent than primary cutaneous B-cell lymphomas [1].

The pathogenesis of CTCL is not fully understood. Clustering CTCL patients have been reported in multiple areas worldwide, including in Sweden [2], Canada [3], Pennsylvania [4] and Texas [5], suggesting a potential environmental exposure. The skin is a barrier organ, and various exposures, including ultraviolet radiation, were described to play a role in the occurrence of malignancies. A mutational profile consistent with ultraviolet B exposure has been identified in CTCL [6], but a causal role of ultraviolet exposure in the occurrence of CTCL has not yet been demonstrated. Several epidemiological studies have been reported [7,8,9,10,11,12,13,14,15,16], but no worldwide meta-analysis has been published to date.

The aim of this study was to describe the epidemiology of CTCL from a global perspective by a systematic review of the literature and meta-analysis. We also aimed to compare the cohorts based on geographic regions to identify a potential spatial clustering of cases, and to analyze the variations of the frequencies of different subgroups of CTCL over time.

## 2. Results

### 2.1. Study Characteristics

**Figure 1:**
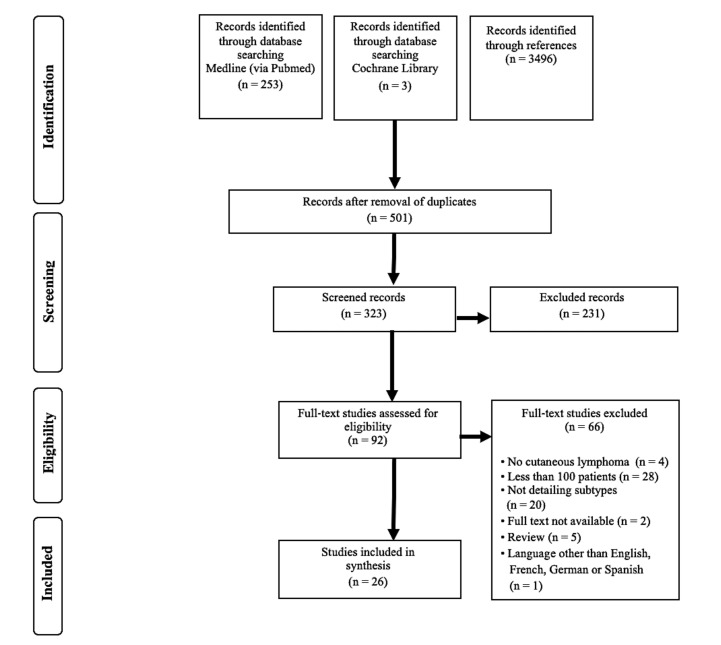
Flowchart of study selection according to the preferred reporting of items for systematic reviews and meta-analyses (PRISMA).

Altogether, 256 publications were identified in the database search, and an additional 3496 records were found in the references of the full-text studies. After the removal of duplicates, 501 articles remained, of which 92 met the inclusion criteria and were read in full. Of these, 26 were included in the meta-analysis. The most frequent reasons for study exclusion were the enrolment of less than 100 patients (1) or the reporting of less than three subtypes of cutaneous lymphomas (2) (detailed in Figure 1). A list of excluded studies is shown in the Table S2, with reasons of exclusion.

The characteristics of included studies are displayed in Table 1 and Figure S1. In total, 16,953 patients from 26 different studies were enrolled in the present meta-analysis. The majority of the studies were reported in European populations; only one South American cohort [7] met the inclusion criteria, and none from Australia or Africa did. The publication dates were evenly distributed over two decades, however there was a gap between 2014 and 2018. The number of included subjects ranged from 120 [11] to 4310 [10]. The study periods varied between four years and decades, and were not precise in two studies [17,18]. Eight reports were considered representative by the authors [10,19,20,21,22,23,24,25]. Many studies from the USA used data from local cancer surveillance registries [10,19,20,21]. The majority of the studies had a prospective inclusion of patients and were multicentric. Only three of them stated a consecutive inclusion of patients. Out of 26 publications, 15 used the World Health Organization- European Organization for Research and Treatment of Cancer (WHO-EORTC) classification; 3 used the WHO [11,19,21] and 3 the EORTC [8,26,27] system, and 3 used a combination of these [8,14,28,29]. In two publications the used classification was not clearly defined [22,30]. Senff et al. [13] reclassified patients using the WHO, EORTC and the WHO-EORTC systems. In total, 15 articles reported the survival of the patients. Grange et al. [26] excluded mycosis fungoides (MF), Sézary syndrome (SS) and lymphomatoid papulosis (LyP) from their report, four studies included CTCL only [21,22,23,29] and three studies reported on CBCL only [13,15,19]. Six publications presented a reporting bias and eight a classification bias.

**Figure 2:**
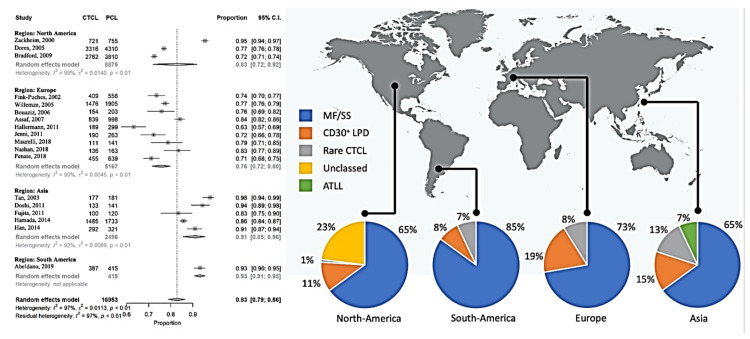
Meta-analysis of the proportion of CTCL compared to all primary cutaneous lymphomas and overview of the subgroups.

Here we report on CTCL only, because there were only minor changes in CTCL between the classification systems. This allowed us to perform a clustering analysis by continents. In total, 83% (95% confidence interval (CI), 70% to 86%) of the primary cutaneous lymphomas were CTCL, which was slightly lower in the European studies and higher in Asian and South American reports (Figure 2). The overall distribution of the CTCL groups varied considerably between the geographic regions (Figure 2).

### 2.2. CTCL Subtypes

#### 2.2.1. Mycosis Fungoides and Sézary Syndrome

**Figure 3:**
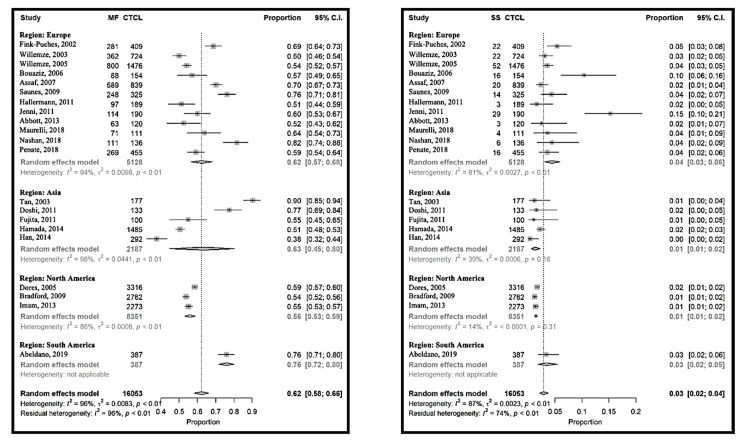
Meta-analyses of the proportion of mycosis fungoides (MF) and Sézary syndrome (SS) compared to CTCL.

MF and SS represented 70% (95% CI 65% to 75%) of the CTCL and seemed to be less frequent in North America (65% (95% CI 55% to 75%)) than in Europe (73% (95% CI 67% to 78%)). The proportion of MF-SS compared to CTCL ranged from 40% (95% CI 34% to 46%) in South Korea [25] to 92% (95% CI 86% to 95%) in Singapore [14], with an important heterogeneity between the studies (Figure S2). Grouping the populations by continent could only explain 1% of this heterogeneity, and even within the continents the studies differed significantly. The same tendency could be observed when focusing only on MF, however here the heterogeneity within the North American studies was lower. The relative frequencies of SS varied between 1% (95% CI 1% to 2%) [21] and 15% (95% CI 10–21%) [12], in the USA and in Switzerland, respectively (Figure 3). Here, more homogeneity could be observed within the continents, and also between them. Grouping by regions could reduce the heterogeneity between the studies by 13%. In summary, MF was the most frequent CTCL, but the MF frequencies varied between the investigated cohorts, and this variation could not be explained by geographical differences. In contrast, the relative frequencies of SS seemed to be globally stable at 3% (95% CI 2% to 4%), and slightly more frequent in Europe. The distribution of MF variants is shown in Figure S3.

#### 2.2.2. CD30-Positive Lymphoproliferative Disorders

CD30-positive LPDs represented 16% (95% CI 13% to 19%) of CTCL, with the lowest in South America at 11% (95% CI 5% to 11%) and the highest in Europe at 19% (95% CI 14% to 24%).

**Figure 4:**
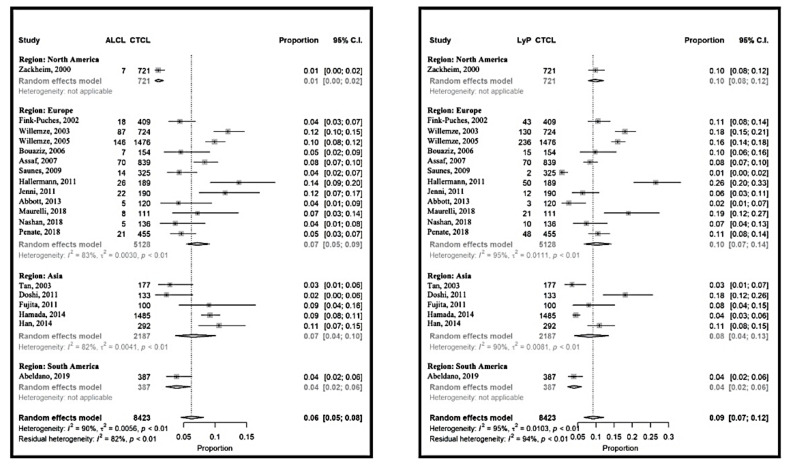
Meta-analyses of the proportions of CD30+ lymphoproliferative disorder subtypes, primary cutaneous anaplastic large cell lymphoma (ALCL) and lymphomatoid papulosis (LyP) compared to CTCL.

The relative frequency of CD30-positive LPDs varied between 6% (95% CI 5% to 7%) [10] and 40% (95% CI 33% to 48%) [32] in the USA and Germany, respectively (Figure 4 and Figure S2). The heterogeneity between the studies was the lowest in Asia, this being 15% (95% CI 11% to 21%). The proportions were more homogeneous for ALCL, at around 7% (95% CI 5% to 9%). The relative frequency of LyP was 9% (95% CI 7% to 12%), with a similar pattern.

#### 2.2.3. Rare Cutaneous T-Cell Lymphomas

**Figure 5:**
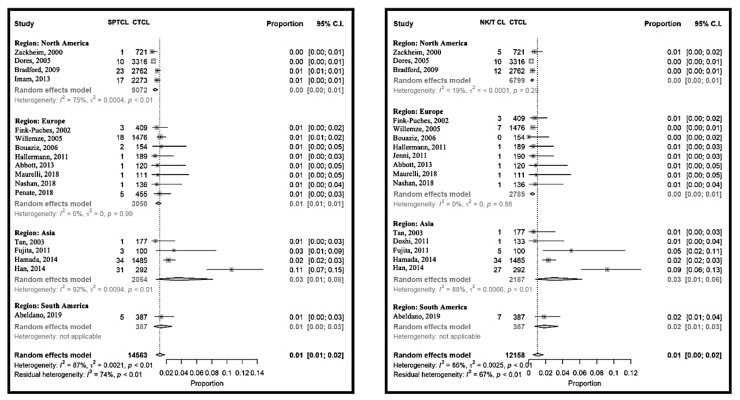
Meta-analyses of the proportion of rare CTCL, subcutaneous panniculitis-like T-cell lymphoma (SPCTL) and NK/T-cell lymphoma compared to CTCL.

The relative frequency of rare cutaneous T-cell lymphomas was 6% (95% CI 4% to 10%) and varied between 1% (95% CI 1% to 2%) [20] and 9% (95% CI 8% to 11%) [31] with the exception of Japan [11,24] and South Korea [25] (Figure 5 and Figure S2). Here, the relative frequencies were 27% (95% CI 19% to 37%) [11] and 39% (95% CI 33% to 45%) [25], respectively. Within the rare CTCL, subcutaneous panniculitis-like lymphoma, NK/T-cell lymphoma (Figure 5) and primary cutaneous T-cell lymphoma not otherwise specified (Figure S4) were more frequent in Asia. Adult T-cell lymphoma and leukemia (ATLL) were not included in the rare cutaneous lymphomas as a group, and were also more frequent in the reports from the Far East (Figure S2). On the other side, other rare lymphomas, including aggressive epidermotropic CD8-positive T-cell lymphoma, primary cutaneous Gamma-Delta T-cell lymphoma and small-medium cell CD4-positive T-cell lymphoma seemed to have similar frequencies around the world. Both within the continents and between the studies, the heterogeneity was importantly lower for each of these latter rare CTCL than for the frequent ones (Figure 5 and Figure S4 ).

## 3. Discussion

In this systematic review and meta-analysis, we reported on the relative frequencies of each CTCL subtype as compared to the total of CTCL cases. We compared these between studies and within continents. A major finding was that CTCL within cutaneous lymphomas seemed to be 15% to 17% more frequent in Asian and South American countries as compared to Europe.

An overall improvement in reporting quality over time could be observed among the studies. From our point of view, it is important to report the time and place of patient recruitment, the use of the current classification system, and to mention if the inclusion is consecutive. Multicentric studies with prospective data collection should be preferred. Although many publications reported on the survival of CTCL patients, a recent systematic review [35] also pointed out the need for new data.

Information on the incidence of CTCL could be derived from some of the studies. Based on selected states, in the USA the incidence was estimated to be 0.77–0.87/100,000 person-years [10,20], whereas others reported an incidence of 0.64/100,000 person-years [36]. In Europe, the incidence was estimated to be 0.29–0.39/100,000 person-years [22,23], with a possible increase over time. In Asia, two studies [24,25] claimed exhaustive records, however none of them reported incidence rates. Due to the heterogeneity of the studies, it was not possible to draw conclusions on CTCL incidence over time. It was estimated in northern American works that the frequency of CTCL increased [37], while Korgavkar et al. [38] suggested a stabilization after this increase in 2013 in the same population. We believe that CTCL are still underdiagnosed, and thereby their number is probably underestimated [39].

The group of MF and SS, representing 70% of the CTCL, revealed an important heterogeneity between and also within the continents. Their lower frequency in North American studies may be explained by the study design in terms of the used registry. Earlier studies did not distinguish between MF and SS [27]; in this case, a separated evaluation of these diseases was not possible in our meta-analysis. It has to be emphasized that the majority of the American data were derived from the Surveillance, Epidemiology, End Results (SEER) program [10,20,21]. The registry used in these publications was not designed explicitly for cutaneous lymphomas, but for malignant diseases in general. Here, cases with uncertain classifications of CTCL subtype may be classed as “other”, including some early MF cases or rare CTCL, explaining the low numbers of MF cases and high numbers of NOS cases. In Asian countries, the proportion of rare CTCL was higher, affecting the MF/SS proportions. While SS represented everywhere 3% of CTCL, the relative frequency of MF was heterogenous. This suggests the influence of environmental factors in the pathogenesis of the latter. The geographical clustering of MF cases, and the occurrence of MF in married couples and families, has been reported in other studies [5], and raises the possibility of an environmental trigger for this malignancy. Lifestyle factors, or industrial exposures [3], may contribute to the pathogenesis of the disease. UV exposure has also been suspected to play a role in the disease pathophysiology [40]. A recent study suggested the role of environmental toxic exposures to benzene and trichloroethylene in the pathogenesis of cutaneous lymphomas [41].

CD30-positive LPDs, especially ALCL, showed less heterogeneity among the studies and continents. Similarly to SS, an innate predisposition of the lymphocytes may explain the occurrence of these diseases, independently from other factors.

Many rare cutaneous lymphomas appeared to be more frequent in Asia, and this was not only true for ATLL, a lymphoma associated with human T-lymphotropic virus (HTLV)-1 infection, which is endemic in Asia. It was proposed that ATLL is similarly frequent in Brazil as compared to Asia [42]. Unfortunately, only one study from South America met our inclusion criteria, while we identified a few reports on lower numbers of patients from Brazil [42,43]. Of note, MF/SS were the most frequent lymphomas, and the CD30-positive LPDs were 11% and 8%, respectively. At the same time, the ATLL accounted for 6% and 22% of CTCL, respectively, in these two excluded Brazilian reports, similarly to the Asian countries. The lowest heterogeneity between studies was observed for Epstein–Barr virus (EBV)-associated NK/T-cell lymphoma and SPTCL, even if these were more frequent in Japan and South Korea. Lymphoma-associated viruses are known to have a higher prevalence in Asia [11,42] and probably in South America. SPTCL has been associated with the presence of germline HAVCR2 mutations, which are more frequent in Asian patients than in European ones [44]. For other rare CTCL, no geographic clustering could be identified in this study.

No publication from China or Africa could be included in this analysis, and some reports from South America and the Near East were excluded. The identified registries from China [45], India [46], Iran [47] or Israel [48] included around 60 patients, and many of them included less than 40 patients, so the comparison of their results is difficult. However, these data would have been interesting, since important differences in CTCL behavior were suggested in pigmented skin [20,49,50]. An additional confounder may be the use of different classification systems. For example, in some publications we were not able to extract the number of SS cases, since they were reported as MF-SS. The histological diagnosis of early MF is difficult, and these cases may have been handled differently between the included studies. There were slight changes between the CTCL classification systems, e.g., the primary cutaneous gamma-delta T-cell lymphoma was previously regarded as SPTCL. ATLL is not included in the EORTC [33] classification, and therefore a minority of the included studies may not have reported it (Figure S1). It is difficult to draw conclusions on trends in CTCL epidemiology over time, since no study reported on CTCL cases within the same geographical area at different timepoints without overlapping, as displayed in Figure S1. Finally, the definition of some lymphomas, such as the primary cutaneous T-cell lymphoma not otherwise specified (PTCL-NOS), has undergone refinement over time. The primary cutaneous gamma-delta T-cell lymphoma was previously regarded as SPTCL. The “SPTCL” subtype in this meta-analysis thus refers to alpha–beta SPTCL, and gamma delta T-cell lymphoma, until the publication by Willemze et al. [31] and the separation of these disease entities in the classification. However, these changes do only slightly affect the results of the meta-analysis. Additionally, the diagnostic strategies and the antibodies used for immunohistochemistry may differ between and even within countries.

Future studies on CTCL epidemiology should use a prospective design to include data on survival. Information is also needed on the staging of lymphomas, including the non-MF/SS types. The inclusion of treatments and outcomes in such registries would help achieve progress in patient care. An independent central review of the included cases could ensure the use of the same classification system and improve the precision of the diagnosis. All these suggestions are already being implemented in the Prospective International Cutaneous Lymphoma Prognostic Index (PROCLIPI) cohort study [39]. However, similar registries are also urgently needed on a national level and for lymphoma subtypes other than MF/SS.

## 4. Materials and Methods

A systematic review and meta-analysis was conducted based on a previously registered and published protocol [51] and according to the preferred reporting of items for systematic reviews and meta-analyses (PRISMA) [52].

### 4.1. Literature Search and Selection

A literature search was conducted based on a standardized strategy [51] in Medline via Pubmed and the Cochrane Library (Table S1). The identified records were screened by two independent reviewers (AM and GD) for eligibility. Reference lists of included studies were screened for additional records. Inclusion criteria were publication in a peer-reviewed journal (1) after 1999 (2) and reporting at least three subtypes (3) of primary cutaneous lymphomas (4) in at least 100 patients (5). Studies focusing on special populations (e.g., children or HIV-infected individuals) were excluded. Only English-, French-, German- or Spanish-language studies were included. The last date of the literature search was 29 July 2019.

### 4.2. Data Extraction and Quality Appraisal

The following information was extracted from the included studies by two independent reviewers: authors, year and title of publication, country and continent of the enrolled patients, time of enrolment, absolute and/or relative numbers of each primary cutaneous lymphoma subtype, staging, survival, used classification system (World Health Organization (WHO) [34], European Organization for Research and Treatment of Cancer (EORTC) [33] or WHO-EORTC [31]) and whether the data were considered representative by the authors. The reporting quality of included studies was rated by two independent reviewers (GD, AM) based on criteria derived from the IHE QA Checklist [53] and rated as present or absent. Items included the prospective/retrospective inclusion of patients, a single center/multicenter cohort and the consecutive inclusion of patients. A reporting bias was considered to be present if multiple CTCL subsets according to the classification were grouped together (e.g., no differentiation between mycosis fungoides (MF) and Sézary syndrome (SS)). Studies with an important number of unclassified cutaneous lymphomas or using various classification systems were considered as having a classification bias. We considered that reporting bias did not affect the cumulative evidence since we handled epidemiologic data of rare diseases.

### 4.3. Statistical Analysis

The absolute numbers of CTCL and CTCL subsets were extracted from the publications. Missing data were calculated within the same publication, if possible. We calculated the numbers in the following three subgroups: MF-SS (classical MF, MF variants and SS), CD30-positive lymphoproliferative disorders (LPD) (anaplastic large cell lymphoma (ALCL) and lymphomatoid papulosis (LyP)), adult T-cell lymphoma/leukemia (ATLL) and rare CTCL (as described by Willemze 2005 [31], including subcutaneous panniculitis-like T-cell lymphoma (SPTCL)). Due to the various numbers of included patients per study, the statistical analyses were performed on relative frequencies (proportions), e.g., cases of MF relative to the total number of CTCL. Proportions were checked for heterogeneity, and were combined using random-effects models to give estimates of the overall proportions. We also investigated whether proportions were consistent across geographic regions. For each region, subgroup proportions were computed. These were then combined across regions using a random effects meta-analysis. To normalize the distributions and to calculate the 95% confidence intervals, the Freeman–Tukey double arcsine transformation was used [54,55]. Statistical analysis was conducted using R Statistics [56] and the Metafor package [57].

## 5. Conclusions

In conclusion, our global meta-analysis of CTCL confirmed the predominance of CTCL among cutaneous lymphomas (83% on average) in the three analyzed continents, most of which were MF cases. It revealed the stability of SS and ALCL frequencies across continents, and the heterogeneity of MF, suggesting the possible role of environmental factors in the pathophysiology of the latter. International studies with a central clinico-histological review of cases, such as that initiated by the Cutaneous Lymphoma International Consortium (PROCLIPI) [58], are extremely valuable in allowing a prospective analysis of CTCL frequencies, outcomes and prognostic factors worldwide. In addition to such international projects, prospective national registries are urgently needed. 

## Figures and Tables

**Table 1:** Characteristics of included studies.

First Author	Year	Country	Inclusion of Patients	*N*	Prospective Data Collection	Multi-Centric	Consecutive	Classification Used	Survival Reported	Reporting Bias	Classifi-Cation Bias
Grange [26]	1999	France	1986–1997	158 ^A^	1	1		EORTC 1997	1	1	
Zackheim [27]	2000	USA	1995–1998	755	1	1		EORTC 1997		1	1
Fink-Puches [28]	2002	Austria	1960–1999	556				EORTC 1997 + WHO			
Tan [14]	2003	Singapore	1990–2001	181				EORTC 1997 + WHO			
Willemze [29]	2003	Netherlands	1985–1999	724 ^B^	1	1		EORTC 1997 + WHO	1		
Dores [10]	2005	USA	1992–2002	4310 ^C^	1	1		WHO-EORTC 2005	1	1	1
Smith [19]	2005	USA	1973–2001	4551 ^C^	1	1		WHO	1		1
Willemze [31]	2005	Austria + Netherlands	1986–2002	1905	1	1		WHO-EORTC 2005	1		
Bouaziz [9]	2006	France	1997–2003	203	1		1	WHO-EORTC 2005	1		
Zinzani [15]	2006	Italy	1980–2003	467 ^B^		1		WHO-EORTC 2005	1		
Assaf [8]	2007	Germany	1999–2007	998	1	1		WHO-EORTC 2005			1
Senff [13]	2007	Netherlands	1985–2005	300 ^B^	1	1		WHO, EORTC 1997, WHO-EORTC 2005	1		
Bradford [20]	2009	USA	2001–2005	3845 ^C^	1	1		WHO-EORTC 2005	1	1	1
Saunes [22]	2009	Norway	1980–2003	325 ^C,D^	1	1		not available			1
Doshi [30]	2011	India	2004–2008	141				not available		1	1
Fujita [11]	2011	Japan	1995–2008	120			1	WHO	1		
Hallermann [32]	2011	Germany	1980–2005	299				WHO-EORTC 2005	1		
Jenni [12]	2011	Switzerland	1990–2009	263				WHO-EORTC 2005	1		
Abbott [23]	2013	United Kingdom	2003–2011	120 ^C,D^	1	1		WHO-EORTC 2005			
Imam [21]	2013	USA	2005–2008	2273 ^C,D^	1	1		WHO	1	1	1
Hamada [24]	2014	Japan	2007–2011	1485 ^C^	1	1		WHO-EORTC 2005			
Han [25]	2014	South-Korea	2006–2010	321 ^C^		1		WHO-EORTC 2005			
Maurelli [16]	2018	Italy	2005–2015	141				WHO-EORTC 2005	1		
Nashan [17]	2018	Germany	2005–2011	163	1			WHO-EORTC 2005			
Penate [18]	2018	Spain	nk–2017	639	1	1		WHO-EORTC 2005	1		
Abeldano [7]	2019	Argentina	2010–2015	415	1	1	1	WHO-EORTC 2005			

nk: not known; ^A^ not reporting MF+LyP; ^B^ only reporting cutaneous B-cell lymphoma (CBCL); ^C^ considered exhaustive by the authors; ^D^ only cutaneous T-cell lymphoma (CTCL); EORTC 1997: European Organisation for Research and Treatment of Cancer 1997 classification [33]; EORTC 1997 + WHO: EORTC 1997 classification completed with elements of the World Health Organisation classification [34]; WHO: World Health Organisation classification [34]; WHO-EORTC 2005: WHO-EORTC 2005 classification [10].

## Supplementary Materials

The following are available online at https://www.mdpi.com/2072-6694/12/10/2921/s1, Table S1: Search strategy, Table S2: Excluded studies with reasons for exclusion, Figure S1: Periods of subject enrolment and used classification systems, Figure S2: Meta-analysis of main CTCL groups compared to CTCL, Figure S3: Meta-analysis of the proportion of mycosis fungoides (MF) variants compared to CTCL, Figure S4: Meta-analysis of the proportion of CTCL, other than MF compared to CTCL.

## References

[1] Willemze R., Cerroni L., Kempf W., Berti E., Facchetti F., Swerdlow S.H., Jaffe E.S. (2019). The 2018 update of the WHO-EORTC classification for primary cutaneous lymphomas. Blood.

[2] Gip L., Nilsson E. (1977). Clustering of mycosis fungoides in the County of Vasternorrland. Lakartidningen.

[3] Ghazawi F.M., Netchiporouk E., Rahme E., Tsang M., Moreau L., Glassman S., Provost N., Gilbert M., Jean S.E., Pehr K. (2017). Comprehensive analysis of cutaneous T-cell lymphoma (CTCL) incidence and mortality in Canada reveals changing trends and geographic clustering for this malignancy. Cancer.

[4] Moreau J.F., Buchanich J.M., Geskin J.Z., Akilov O.E., Geskin L.J. (2014). Non-random geographic distribution of patients with cutaneous T-cell lymphoma in the Greater Pittsburgh Area. Dermatol. Online J..

[5] Litvinov I.V., Tetzlaff M.T., Rahme E., Habel Y., Risser D.R., Gangar P., Jennings M.A., Pehr K., Prieto V.G., Sasseville D. (2015). Identification of geographic clustering and regions spared by cutaneous T-cell lymphoma in Texas using 2 distinct cancer registries. Cancer.

[6] Wang L., Ni X., Covington K.R., Yang B.Y., Shiu J., Zhang X., Xi L., Meng Q., Langridge T., Drummond J. (2015). Genomic profiling of Sezary syndrome identifies alterations of key T cell signaling and differentiation genes. Nat. Genet..

[7] Abeldano A., Enz P., Maskin M., Cervini A.B., Torres N., Acosta A.C., Narbaitz M., Vanzulli S., Orentrajch M., Villareal M.A. (2019). Primary cutaneous lymphoma in Argentina: A report of a nationwide study of 416 patients. Int. J. Dermatol..

[8] Assaf C., Gellrich S., Steinhoff M., Nashan D., Weisse F., Dippel E., Coors E., Stein A., Gollin P., Henke U. (2007). Cutaneous lymphomas in Germany: An analysis of the Central Cutaneous Lymphoma Registry of the German Society of Dermatology (DDG). J. Dtsch. Dermatol. Ges..

[9] Bouaziz J.D., Bastuji-Garin S., Poszepczynska-Guigne E., Wechsler J., Bagot M. (2006). Relative frequency and survival of patients with primary cutaneous lymphomas: Data from a single-centre study of 203 patients. Br. J. Dermatol..

[10] Dores G.M., Anderson W.F., Devesa S.S. (2005). Cutaneous lymphomas reported to the National Cancer Institute’s surveillance, epidemiology, and end results program: Applying the new WHO-European Organisation for Research and Treatment of Cancer classification system. J. Clin. Oncol..

[11] Fujita A., Hamada T., Iwatsuki K. (2011). Retrospective analysis of 133 patients with cutaneous lymphomas from a single Japanese medical center between 1995 and 2008. J. Dermatol..

[12] Jenni D., Karpova M.B., Seifert B., Golling P., Cozzio A., Kempf W., French L.E., Dummer R. (2011). Primary cutaneous lymphoma: Two-decade comparison in a population of 263 cases from a Swiss tertiary referral centre. Br. J. Dermatol..

[13] Senff N.J., Hoefnagel J.J., Jansen P.M., Vermeer M.H., van Baarlen J., Blokx W.A., Canninga-van Dijk M.R., Geerts M.L., Hebeda K.M., Kluin P.M. (2007). Reclassification of 300 primary cutaneous B-Cell lymphomas according to the new WHO-EORTC classification for cutaneous lymphomas: Comparison with previous classifications and identification of prognostic markers. J. Clin. Oncol..

[14] Tan S.H., Sim C.S., Ong B.H. (2003). Cutaneous lymphomas other than mycosis fungoides in Singapore: A clinicopathological analysis using recent classification systems. Br. J. Dermatol..

[15] Zinzani P.L., Quaglino P., Pimpinelli N., Berti E., Baliva G., Rupoli S., Martelli M., Alaibac M., Borroni G., Chimenti S. (2006). Prognostic factors in primary cutaneous B-cell lymphoma: The Italian Study Group for Cutaneous Lymphomas. J. Clin. Oncol..

[16] Maurelli M., Tessari G., Colato C., Schena D., Girolomoni G. (2018). Incidence and ten-year follow-up of primary cutaneous lymphomas: A single-centre cohort study. Eur. J. Dermatol..

[17] Nashan D., Friedrich C.M., Geissler E., Schmitt-Graeff A., Klein F., Meiss F. (2018). Primary cutaneous lymphoma-a case series of 163 patients. Hautarzt.

[18] Penate Y., Servitje O., Machan S., Fernandez-de-Misa R., Estrach M.T., Acebo E., Mitxelena J., Ramon M.D., Florez A., Blanes M. (2018). The First Year of the AEVD Primary Cutaneous Lymphoma Registry. Actas Dermo-Sifiliogr..

[19] Smith B.D., Smith G.L., Cooper D.L., Wilson L.D. (2005). The cutaneous B-cell lymphoma prognostic index: A novel prognostic index derived from a population-based registry. J. Clin. Oncol..

[20] Bradford P.T., Devesa S.S., Anderson W.F., Toro J.R. (2009). Cutaneous lymphoma incidence patterns in the United States: A population-based study of 3884 cases. Blood.

[21] Imam M.H., Shenoy P.J., Flowers C.R., Phillips A., Lechowicz M.J. (2013). Incidence and survival patterns of cutaneous T-cell lymphomas in the United States. Leuk. Lymphoma.

[22] Saunes M., Nilsen T.I., Johannesen T.B. (2009). Incidence of primary cutaneous T-cell lymphoma in Norway. Br. J. Dermatol..

[23] Abbott R.A., Aldridge C., Dojcinov S., Piguet V. (2013). Incidence of primary cutaneous T-cell lymphoma in Wales. Br. J. Dermatol..

[24] Hamada T., Iwatsuki K. (2014). Cutaneous lymphoma in Japan: A nationwide study of 1733 patients. J. Dermatol..

[25] Han J.H., Ko Y.H., Kang Y.K., Kim W.S., Kim Y.J., Kim I., Kim H.J., Min S.K., Park C.K., Park C.S. (2014). Characteristics of Cutaneous Lymphomas in Korea According to the New WHO-EORTC Classification: Report of a Nationwide Study. Korean J. Pathol..

[26] Grange F., Hedelin G., Joly P., Beylot-Barry M., D’Incan M., Delaunay M., Vaillant L., Avril M.F., Bosq J., Wechsler J. (1999). Prognostic factors in primary cutaneous lymphomas other than mycosis fungoides and the Sezary syndrome. The French Study Group on Cutaneous Lymphomas. Blood.

[27] Zackheim H.S., Vonderheid E.C., Ramsay D.L., LeBoit P.E., Rothfleisch J., Kashani-Sabet M. (2000). Relative frequency of various forms of primary cutaneous lymphomas. J. Am. Acad. Dermatol..

[28] Fink-Puches R., Zenahlik P., Back B., Smolle J., Kerl H., Cerroni L. (2002). Primary cutaneous lymphomas: Applicability of current classification schemes (European Organization for Research and Treatment of Cancer, World Health Organization) based on clinicopathologic features observed in a large group of patients. Blood.

[29] Willemze R. (2003). Cutaneous T-cell lymphoma: Epidemiology, etiology, and classification. Leuk. Lymphoma.

[30] Doshi B.R., Khopkar U.S. (2011). Retrospective study of spectrum of cutaneous lymphoma presenting to dermatology. Indian J. Derm. Venereol. Leprol..

[31] Willemze R., Jaffe E.S., Burg G., Cerroni L., Berti E., Swerdlow S.H., Ralfkiaer E., Chimenti S., Diaz-Perez J.L., Duncan L.M. (2005). WHO-EORTC classification for cutaneous lymphomas. Blood.

[32] Hallermann C., Niermann C., Fischer R.J., Schulze H.J. (2011). Survival data for 299 patients with primary cutaneous lymphomas: A monocentre study. Acta Derm. Venereol..

[33] Willemze R., Kerl H., Sterry W., Berti E., Cerroni L., Chimenti S., Diaz-Perez J.L., Geerts M.L., Goos M., Knobler R. (1997). EORTC classification for primary cutaneous lymphomas: A proposal from the Cutaneous Lymphoma Study Group of the European Organization for Research and Treatment of Cancer. Blood.

[34] Harris N.L., Jaffe E.S., Diebold J., Flandrin G., Muller-Hermelink H.K., Vardiman J., Lister T.A., Bloomfield C.D. (2000). The World Health Organization classification of hematological malignancies report of the Clinical Advisory Committee Meeting, Airlie House, Virginia, November 1997. Mod. Pathol..

[35] Mourad A., Gniadecki R. (2020). Overall Survival in Mycosis Fungoides: A Systematic Review and Meta-Analysis. J. Investig. Dermatol..

[36] Wilson L.D., Hinds G.A., Yu J.B. (2012). Age, race, sex, stage, and incidence of cutaneous lymphoma. Clin. Lymphoma Myeloma Leuk..

[37] Criscione V.D., Weinstock M.A. (2007). Incidence of cutaneous T-cell lymphoma in the United States, 1973–2002. Arch. Dermatol..

[38] Korgavkar K., Xiong M., Weinstock M. (2013). Changing incidence trends of cutaneous T-cell lymphoma. JAMA Dermatol..

[39] Scarisbrick J.J., Quaglino P., Prince H.M., Papadavid E., Hodak E., Bagot M., Servitje O., Berti E., Ortiz-Romero P., Stadler R. (2019). The PROCLIPI international registry of early-stage mycosis fungoides identifies substantial diagnostic delay in most patients. Br. J. Dermatol..

[40] DeStefano C.B., Desale S., Fernandez S.J., Shenoy A.G. (2019). The impact of environmental ultraviolet exposure on the clinical course of mycosis fungoides. J. Am. Acad. Dermatol..

[41] Clough L., Bayakly A.R., Ward K.C., Khan M.K., Chen S.C., Lechowicz M.J., Flowers C.R., Allen P.B., Switchenko J.M. (2020). Clustering of cutaneous T-cell lymphoma is associated with increased levels of the environmental toxins benzene and trichloroethylene in the state of Georgia. Cancer.

[42] Nudelmann L.M., Bonamigo R.R. (2015). Primary cutaneous lymphoma in southern Brazil: A 12-year single-center experience. Int. J. Dermatol..

[43] Bittencourt A.L., Oliveira P.D., Andrade A.C., Santos T.C., Oliveira R.F., Farre L., Araujo I. (2013). Analysis of cutaneous lymphomas in a medical center in Bahia, Brazil. Am. J. Clin. Pathol..

[44] Sonigo G., Battistella M., Beylot-Barry M., Ingen-Housz-Oro S., Franck N., Barete S., Boulinguez S., Dereure O., Bonnet N., Socie G. (2020). HAVCR2 mutations are associated with severe hemophagocytic syndrome in subcutaneous panniculitis-like T-cell lymphoma. Blood.

[45] Au W.Y., Ma S.Y., Chim C.S., Choy C., Loong F., Lie A.K., Lam C.C., Leung A.Y., Tse E., Yau C.C. (2005). Clinicopathologic features and treatment outcome of mature T-cell and natural killer-cell lymphomas diagnosed according to the World Health Organization classification scheme: A single center experience of 10 years. Ann. Oncol..

[46] Naresh K.N., Srinivas V., Soman C.S. (2000). Distribution of various subtypes of non-Hodgkin’s lymphoma in India: A study of 2773 lymphomas using R.E.A.L. and WHO Classifications. Ann. Oncol..

[47] Manuchehri H.M., Rakhshan M. (2006). Characteristics of primary cutaneous lymphomas in Tehran, Iran (1998–2004). J. Eur. Acad. Dermatol. Venereol..

[48] Khamaysi Z., Ben-Arieh Y., Izhak O.B., Epelbaum R., Dann E.J., Bergman R. (2008). The applicability of the new WHO-EORTC classification of primary cutaneous lymphomas to a single referral center. Am. J. Dermatopathol..

[49] Geller S., Lebowitz E., Pulitzer M.P., Horwitz S.M., Moskowitz A.J., Dusza S., Myskowski P.L. (2019). Outcomes and prognostic factors in African American and black patients with mycosis fungoides/Sezary syndrome: Retrospective analysis of 157 patients from a referral cancer center. J. Am. Acad. Dermatol..

[50] Adams S.V., Newcomb P.A., Shustov A.R. (2016). Racial Patterns of Peripheral T-Cell Lymphoma Incidence and Survival in the United States. J. Clin. Oncol..

[51] Dobos G., Bagot M., de Masson A. Epidemiology of Cutaneous Lymphomas: A Systematic Review of Relative Frequencies. https://www.crd.york.ac.uk/prospero/display_record.php?ID=CRD42020148295..

[52] Moher D., Liberati A., Tetzlaff J., Altman D.G., Group P. (2009). Preferred reporting items for systematic reviews and meta-analyses: The PRISMA statement. J. Clin. Epidemiol..

[53] Guo B., Moga C., Harstall C., Schopflocher D. (2016). A principal component analysis is conducted for a case series quality appraisal checklist. J. Clin. Epidemiol..

[54] Freeman M.F., Tukey J.W. (1950). Transformations related to the angular and the square root. Ann. Math. Stat..

[55] Miller J.J. (1978). The inverse of the Freeman-Tukey double arcsine transformation. Am. Stat..

[56] Team, R.C. R: A Language and Environment for Statistical Computing. http://www.R-project.org/.

[57] Viechtbauer W. (2010). Conducting meta-analyses in R with the metafor package. J. Stat. Softw..

[58] Scarisbrick J.J., Prince H.M., Vermeer M.H., Quaglino P., Horwitz S., Porcu P., Stadler R., Wood G.S., Beylot-Barry M., Pham-Ledard A. (2015). Cutaneous Lymphoma International Consortium Study of Outcome in Advanced Stages of Mycosis Fungoides and Sezary Syndrome: Effect of Specific Prognostic Markers on Survival and Development of a Prognostic Model. J. Clin. Oncol..

