# Supplementary material for: Epidemiology of Cutaneous T-Cell Lymphomas: A Systematic Review and Meta-Analysis of 16,953 Patients
Source: Cancers (Basel). 2020 Oct 11;12(10):2921. doi: 10.3390/cancers12102921 (PMC7600606; doi:10.3390/cancers12102921)
Supplement: Supplementary file 1 [file cancers-12-02921-s001.pdf]

Table S1. Search strategy used on Medline database via Pubmed

Order	Used search terms
1.	inciden*
2.	prevalen*
3.	#1 or #2
4.	primary cutaneous lymphoma
5.	#3 and #4

Table S2. List of excluded studies with reasons of exclusion

Authors	Year	Title	Journal	Reference Volume (Issue): Pages	Reasons for exclusion
Beylot-Barry, M., B. Vergier, et al.	1999	The Spectrum of Cutaneous Lymphomas in HIV infection: a study of 21 cases	Am J Surg Pathol	23(10): 1208-1216.	selected population, <100
Martin Carrasco, P., M. Morillo Andujar, et al.	2016	[Primary cutaneous B-cell lymphomas: study of 22 cases]	Med Clin (Barc)	147(5): 207-210.	<100, 22 CBCL
Manuchehri, H. M. and M. Rakhshan	2006	Characteristics of primary cutaneous lymphomas in Tehran, Iran (1998-2004)	J Eur Acad Dermatol Venereol	20(6): 758-760.	<100, 25, Teheran
Nagasawa, T., H. Miwa, et al.	2000	Characteristics of cutaneous lymphomas in Osaka, Japan (1988-1999) based on the European Organization for Research and Treatment of Cancer classification	Am J Dermatopathol	22(6): 510-514.	<100, 25, Japan
Au, W. Y., S. Y. Ma, et al.	2005	Clinicopathologic features and treatment outcome of mature T-cell and natural killer-cell lymphomas diagnosed according to the World Health Organization classification scheme: a single center experience of 10 years	Ann Oncol	16(2): 206-214.	<100, 27, China
Naresh, K. N., V. Srinivas, et al.	2000	Distribution of various subtypes of non-Hodgkin's lymphoma in India: a study of 2773 lymphomas using R.E.A.L. and WHO Classifications	Ann Oncol	11 Suppl 1: 63-67.	<100, 31, India
Smith, B. D., E. J. Glusac, et al.	2004	Primary cutaneous B-cell lymphoma treated with radiotherapy: a comparison of the European Organization for Research and Treatment of Cancer and the WHO classification systems	J Clin Oncol	22(4): 634-639.	<100, 34, USA
Savage, K. J., M. Chhanabhai, et al.	2004	Characterization of peripheral T-cell lymphomas in a single North American institution by the WHO classification	Ann Oncol	15(10): 1467-1475.	<100, 43, Canada
Khamaysi, Z., Y. Ben-Arieh, et al.	2008	The applicability of the new WHO-EORTC classification of primary cutaneous lymphomas to a single referral center	Am J Dermatopathol	30(1): 37-44.	<100, 43, Israel
	2000	The world health organization classification of malignant lymphomas in japan: incidence of recently recognized entities. Lymphoma Study Group of Japanese Pathologists	Pathol Int	50(9): 696-702.	<100, 47, japan
Lee, M. W. and G. Korean Dermatopathology Research	2003	Characteristics of cutaneous lymphomas in Korea	Clin Exp Dermatol	28(6): 639-646.	<100, 49, Korea
Al Diab, A. R., A. Aleem, et al.	2011	Clinico-pathological pattern of extranodal non-Hodgkin's lymphoma in Saudi Arabia	Asian Pac J Cancer Prev	12(12): 3277-3282.	<100, 51, not detailing subtypes
Cieza-Diaz, D. E., E. Conde-Montero, et al.	2017	Epidemiological and clinical features of patients diagnosed with cutaneous T-cell lymphomas in a Spanish tertiary care hospital	J Eur Acad Dermatol Venereol	31(3): e150-e153.	<100, 53, Spain
Liao, J. B., S. S. Chuang, et al.	2010	Clinicopathologic analysis of cutaneous lymphoma in taiwan: a high frequency of extranodal natural killer/t-cell lymphoma, nasal type, with an extremely poor prognosis	Arch Pathol Lab Med	134(7): 996-1002.	<100, 56, Taiwan
Burad, D. K., M. M. Therese, et al.	2012	Peripheral T-cell lymphoma: frequency and distribution in a tertiary referral center in South India	Indian J Pathol Microbiol	55(4): 429-432.	<100, 61, India
Bessell, E. M., C. E. Humber, et al.	2012	Primary cutaneous B-cell lymphoma in Nottinghamshire U.K.: prognosis of subtypes defined in the WHO-EORTC classification	Br J Dermatol	167(5): 1118-1123.	<100, 61, UK
Yasukawa, K., N. Kato, et al.	2006	The spectrum of cutaneous lymphomas in Japan: a study of 62 cases based on the World Health Organization Classification	J Cutan Pathol	33(7): 487-491.	<100, 62, Japan
Lee, M. Y., M. H. Tsou, et al.	2005	Clinicopathological analysis of T-cell lymphoma in Taiwan according to WHO classification: high incidence of enteropathy-type intestinal T-cell lymphoma	Eur J Haematol	75(3): 221-226.	<100, 7, Korea
Lee, M., T. Tan, et al.	2006	Clinicopathological analysis of 598 malignant lymphomas in Taiwan: seven-year experience in a single institution	Am J Pathol	81(8): 568-575.	<100, 7, Taiwan
Riou-Gotta, M. O., E. Fournier, et al.	2008	Primary cutaneous lymphomas: a population-based descriptive study of 71 consecutive cases diagnosed between 1980 and 2003	Leuk Lymphoma	49(8): 1537-1544.	<100, 71
Goteri, G., R. Ranaldi, et al.	2007	Clinicopathological features of primary cutaneous B-cell lymphomas from an academic regional hospital in central Italy: no evidence of Borrelia burgdorferi association	Leuk Lymphoma	48(11): 2184-2188.	<100, 73, Italy
Bittencourt, A. L., P. D. Oliveira, et al.	2013	Analysis of cutaneous lymphomas in a medical center in Bahia, Brazil	Am J Clin Pathol	140(3): 348-354.	<100, 85, Brazil
Eder, J., A. Kern, et al.	2015	Frequency of primary cutaneous lymphoma variants in Austria: retrospective data from a dermatology referral centre between 2006 and 2013	J Eur Acad Dermatol Venereol	29(8): 1517-1523.	<100, 86, Austria
Vilar Coromina, N. and R. Marcos-Gragera	2008	[Primary cutaneous lymphomas. Population-based incidence and survival, 1994-2004]	Med Clin (Barc)	131(10): 396-397.	<100, 86, Spain
Nudelmann, L. M. and R. R. Bonamigo	2015	Primary cutaneous lymphoma in southern Brazil: a 12-year single-center experience	Int J Dermatol	54(12): e512-520.	<100, 89, Brazil
Lee, J. H., J. H. Lee, et al.	2013	Characteristics of primary cutaneous lymphoma according to WHO-EORTC classification in Korea	Clin Exp Dermatol	38(5): 457-463.	<100, 93, South-Korea
Park, J. H., H. T. Shin, et al.	2012	World Health Organization-European Organization for Research and Treatment of Cancer classification of cutaneous lymphoma in Korea: a retrospective study at a single tertiary institution	J Am Acad Dermatol	67(6): 1200-1209.	<100, 96, Korea
Ponzoni, M., A. J. Ferreri, et al.	2011	Prevalence of Borrelia burgdorferi infection in a series of 98 primary cutaneous lymphomas	Oncologist	16(11): 1582-1588.	<100, 98, Italy
Gaitonde, S., S. Kavuri, et al.	2008	EBV positivity in primary cutaneous large B-cell lymphoma with immunophenotypic features of leg type: an isolated incident or something more significant?	Acta Oncol	47(3): 461-464.	<100, casereport
Cen, X., M. Ma, et al.	2003	[Primary extranodal lymphomas: analyses of clinical characteristics and misdiagnoses of 139 cases]	Beijing Da Xue Xue Bao Yi Xue Ban	35(2): 143-145.	Chinese language
Senff, N. J., J. J. Hoefnagel, et al.	2007	Results of radiotherapy in 153 primary cutaneous B-Cell lymphomas classified according to the WHO-EORTC classification	Arch Dermatol	143(12): 1520-1526.	duplicata
Niitsu, N., M. Okamoto, et al.	2008	Clinico-pathologic features and outcome of Japanese patients with peripheral T-cell lymphomas	Hematol Oncol	26(3): 152-158.	not cutaneous lymphoma
Ohshima, K., J. Suzumiya, et al.	2002	The World Health Organization classification of malignant lymphoma: incidence and clinical prognosis in HTLV-1-endemic area of Fukuoka	Pathol Int	52(1): 1-12.	not cutaneous lymphoma
Mitterlechner, T., M. Fiegl, et al.	2006	Epidemiology of non-Hodgkin lymphomas in Tyrol/Austria from 1991 to 2000	J Clin Pathol	59(1): 48-55.	not cutaneous lymphomas
Kim, Y. J., H. J. Shin, et al.	2018	The Incidence of Other Primary Cancers in Patients with Cutaneous Lymphoma	Ann Dermatol	30(3): 335-341.	not cutaneous lymphomas
Brownell, I., C. J. Etzel, et al.	2008	Increased malignancy risk in the cutaneous T-cell lymphoma patient population	Clin Lymphoma Myeloma	8(2): 100-105.	not detailing subtypes
van Leeuwen, M. T., J. J. Turner, et al.	2013	Latitude gradients for lymphoid neoplasm subtypes in Australia support an association with ultraviolet radiation exposure	Int J Cancer	133(4): 944-951.	not detailing subtypes
Amber, K. T., R. Bloom, et al.	2016	Second Primary Malignancies in CTCL Patients from 1992 to 2011: A SEER-Based, Population-Based Study Evaluating Time from CTCL Diagnosis, Age, Sex, Stage, and CD30+ Subtype	Am J Clin Dermatol	17(1): 71-77.	not detailing subtypes
Groves, F. D., M. S. Linet, et al.	2000	Cancer surveillance series: non-Hodgkin's lymphoma incidence by histologic subtype in the United States from 1978 through 1995	J Natl Cancer Inst	92(15): 1240-1251.	not detailing subtypes
Vakeva, L., E. Pukkala, et al.	2000	Increased risk of secondary cancers in patients with primary cutaneous T cell lymphoma	J Invest Dermatol	115(1): 62-65.	not detailing subtypes
Economopoulos, T., S. Papageorgiou, et al.	2005	Non-Hodgkin's lymphomas in Greece according to the WHO classification of lymphoid neoplasms. A retrospective analysis of 810 cases	Acta Haematol	113(2): 97-103.	not detailing subtypes
Stang, A., B. Streller, et al.	2006	Incidence of skin lymphoma in Germany	Ann Epidemiol	16(3): 214-222.	not detailing subtypes

Figure S1. Periods of subject enrolment and used classification systems

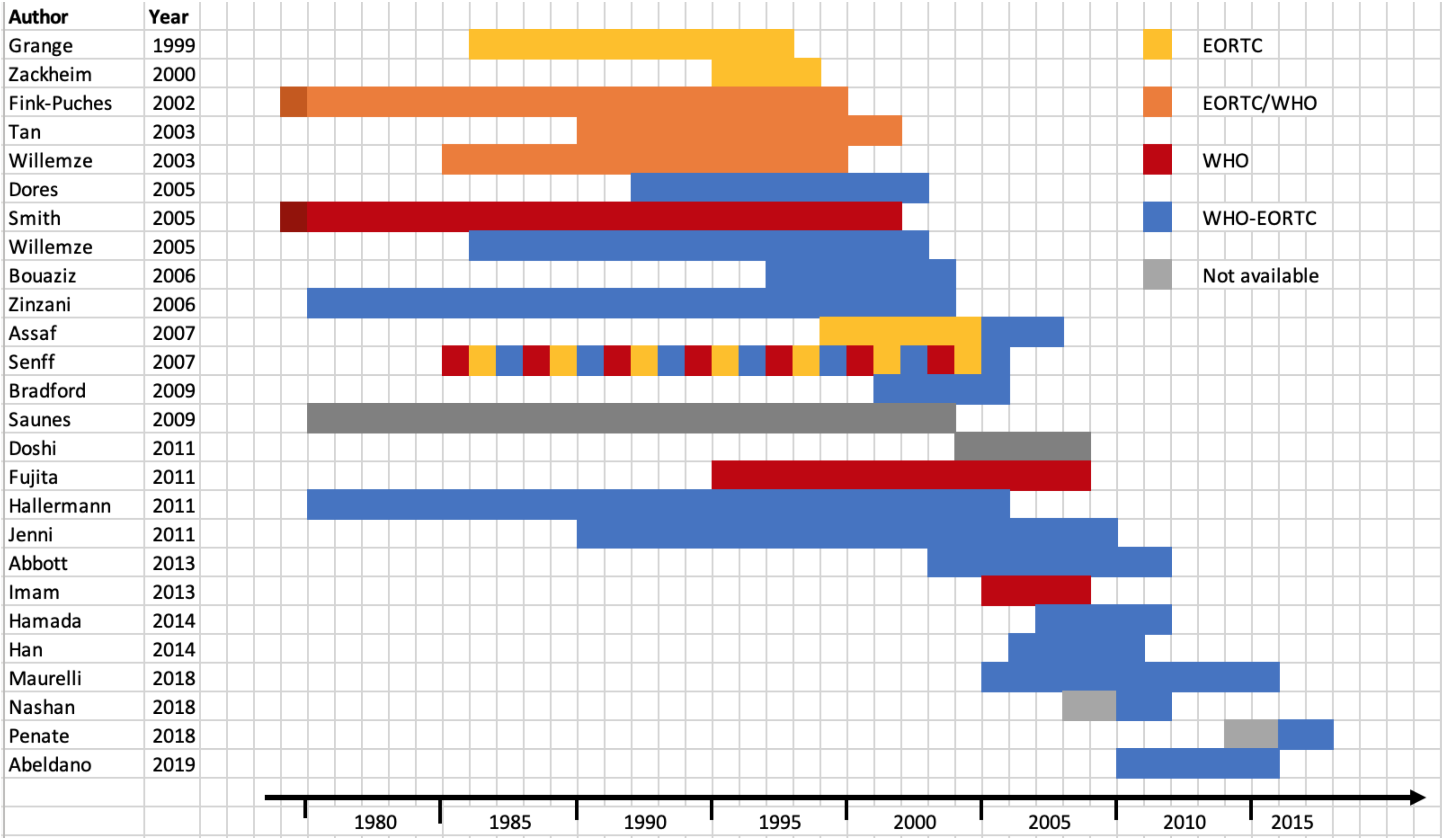

EORTC: European Organisation for Research and Treatment of Cancer; WHO: World Health Organisation

Figure S2. Meta-analysis of the proportion of main CTCL groups compared to CTCL

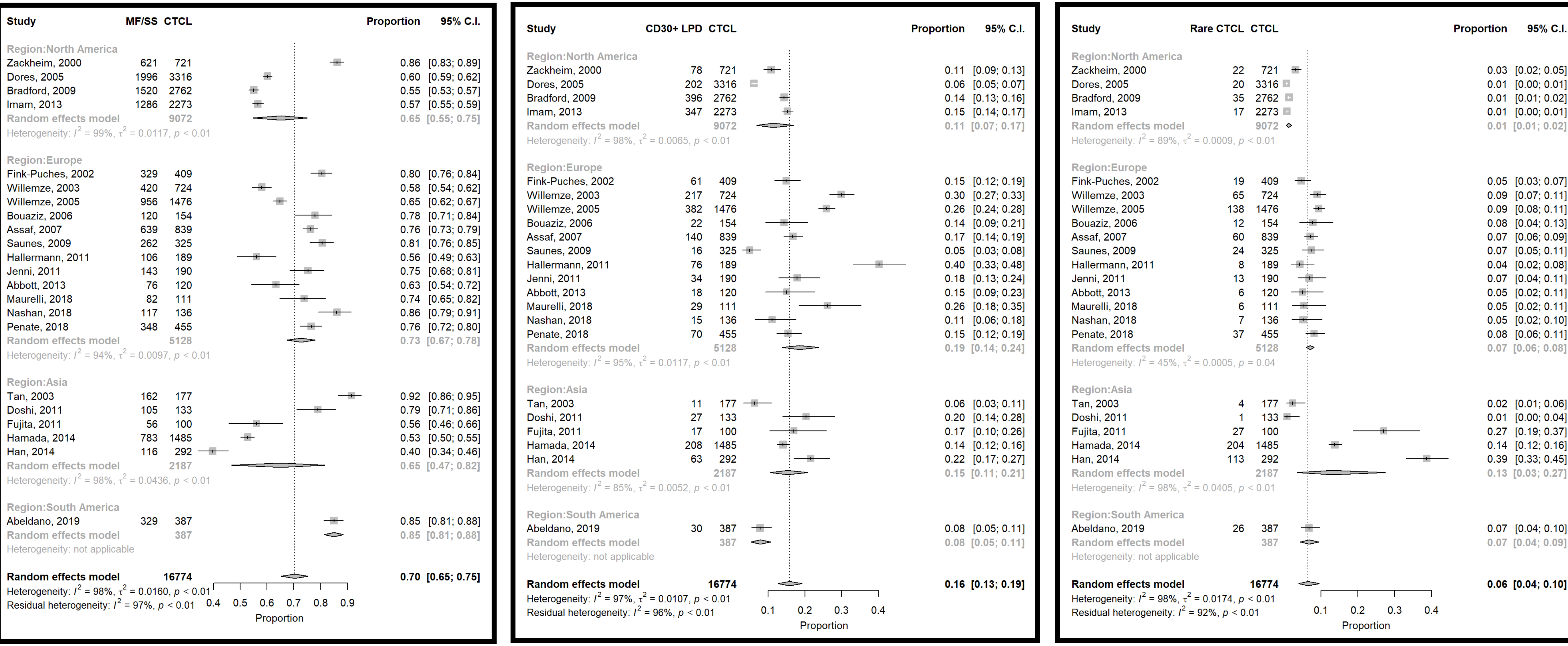

CTCL: cutaneous T-cell lymphomas; MF/SS: mycosis fungoides and S ézary syndrome; CD30+ LPD: CD30-positive lymphoproliferative disorders

Figure S3. Meta-analysis of the proportion of mycosis fungoides (MF) variants compared to CTCL

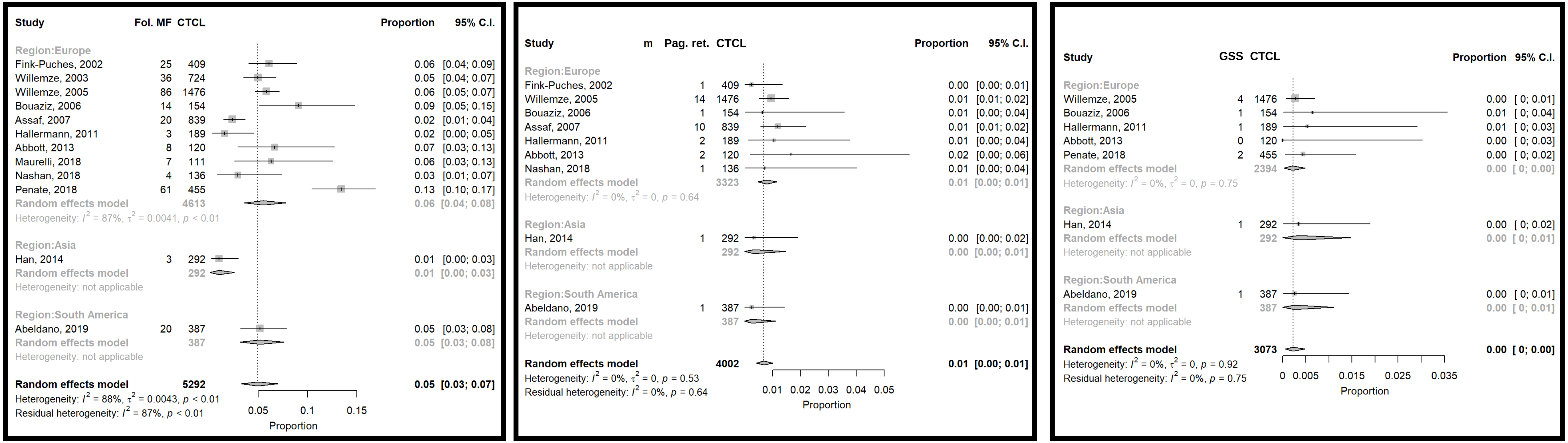

Fol. MF: folliculotropic MF; Pag. ret.: Pagetoid reticulosis; GSS: granulomatous slack skin

Figure S4/a. Meta-analysis of the proportion of CTCL, other than MF compared to CTCL

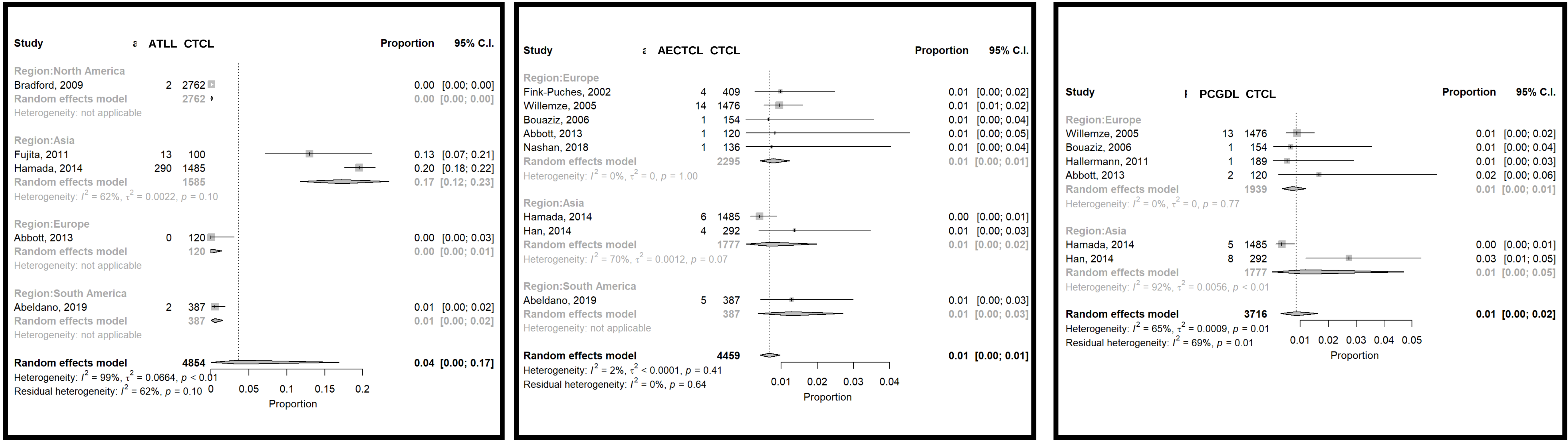

ATLL: adultd T-cell lymphoma/leukemia; AECTCL: aggressive epidermotropic CD8-positive T-cell lymphoma; PCGDL: primary cutaneous gamma-delta-cell lymphoma

Figure S4/b. Meta-analysis of the proportion of CTCL, other than MF compared to CTCL

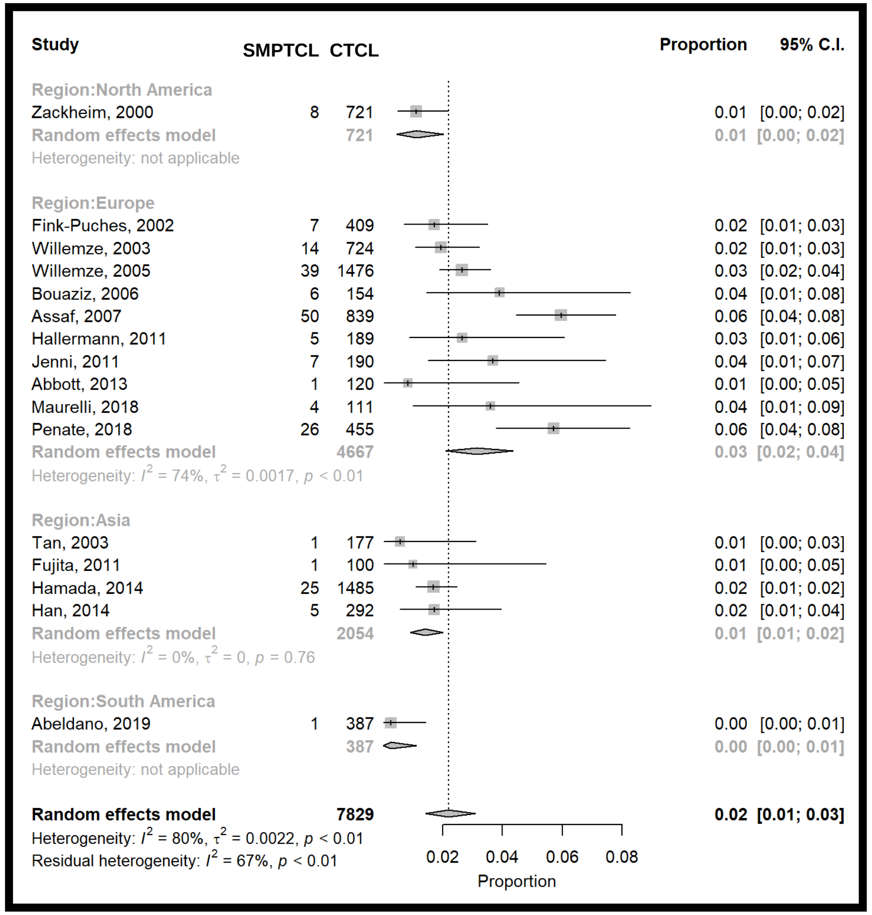

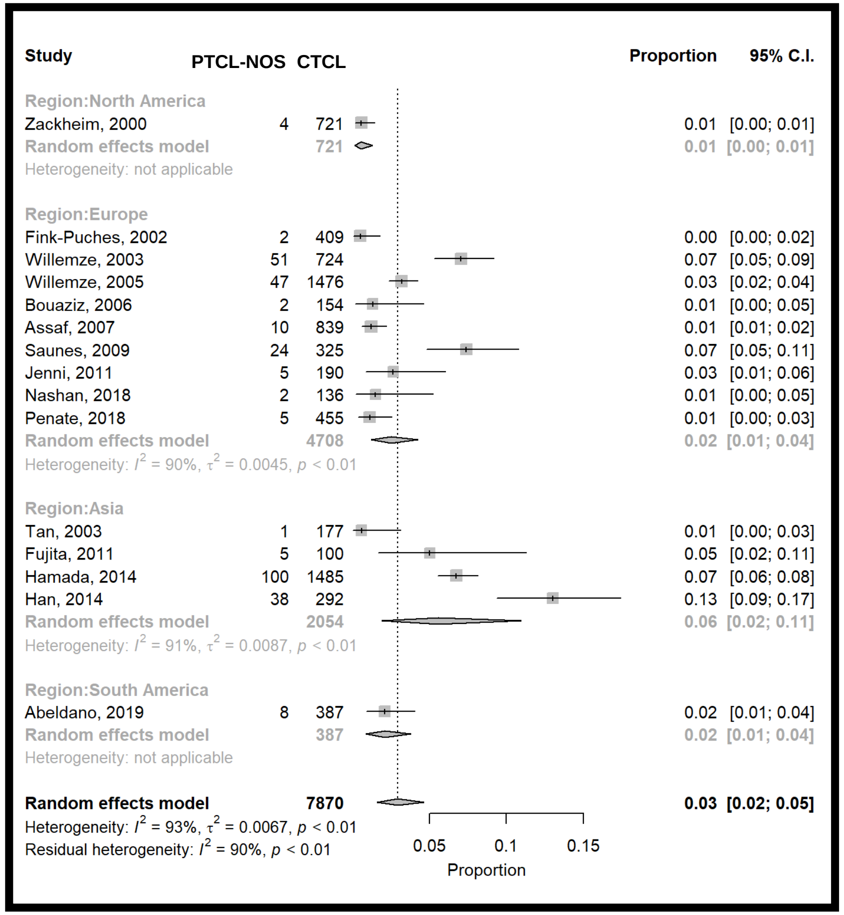

SMPTCL: CD4-positive small/medium cell lymphoproliferation; PTCL-NOS: primary cutaneous T-cell lymphoma not other specified
